# Supplementary material for: The online metacognitive control of decisions
Source: Commun Psychol. 2024 Mar 27;2:23. doi: 10.1038/s44271-024-00071-y (PMC11332065; doi:10.1038/s44271-024-00071-y)
Supplement: Supplementary file 3 — Reporting Summary [file 44271_2024_71_MOESM3_ESM.pdf]

nature portfolio

Corresponding author(s): Jean Daunizeau

Last updated by author(s): May 10, 2023

Reporting Summary

Nature Portfolio wishes to improve the reproducibility of the work that we publish. This form provides structure for consistency and transparency in reporting. For further information on Nature Portfolio policies, see our Editorial Policies and the Editorial Policy Checklist.

Statistics

For all statistical analyses, confirm that the following items are present in the figure legend, table legend, main text, or Methods section.

n/a	Confirmed	
☐	☒	The exact sample size (*n*) for each experimental group/condition, given as a discrete number and unit of measurement
☐	☒	A statement on whether measurements were taken from distinct samples or whether the same sample was measured repeatedly
☒	☐	The statistical test(s) used AND whether they are one- or two-sided<br>*Only common tests should be described solely by name; describe more complex techniques in the Methods section.*
☐	☒	A description of all covariates tested
☒	☐	A description of any assumptions or corrections, such as tests of normality and adjustment for multiple comparisons
☒	☐	A full description of the statistical parameters including central tendency (e.g. means) or other basic estimates (e.g. regression coefficient) AND variation (e.g. standard deviation) or associated estimates of uncertainty (e.g. confidence intervals)
☒	☐	For null hypothesis testing, the test statistic (e.g. *F*, *t*, *r*) with confidence intervals, effect sizes, degrees of freedom and *P* value noted<br>*Give P values as exact values whenever suitable.*
☒	☐	For Bayesian analysis, information on the choice of priors and Markov chain Monte Carlo settings
☒	☐	For hierarchical and complex designs, identification of the appropriate level for tests and full reporting of outcomes
☒	☐	Estimates of effect sizes (e.g. Cohen's *d*, Pearson's *r*), indicating how they were calculated

Our web collection on statistics for biologists contains articles on many of the points above.

Software and code

Policy information about availability of computer code

Data collection	Behavioral measures obtained with Psychtoolbox (http://psychtoolbox.org/)
Data analysis	All data analyses were performed using the VBA freeware (https://mbb-team.github.io/VBA-toolbox/)

For manuscripts utilizing custom algorithms or software that are central to the research but not yet described in published literature, software must be made available to editors and reviewers. We strongly encourage code deposition in a community repository (e.g. GitHub). See the Nature Portfolio guidelines for submitting code & software for further information.

Data

Policy information about availability of data

All manuscripts must include a data availability statement. This statement should provide the following information, where applicable:

- Accession codes, unique identifiers, or web links for publicly available datasets
- A description of any restrictions on data availability
- For clinical datasets or third party data, please ensure that the statement adheres to our policy

All empirical data (as well as analysis code) is available here: https://owncloud.icm-institute.org/index.php/s/wAsSPNndwZVlBlR.

Human research participants

Policy information about studies involving human research participants and Sex and Gender in Research.

Reporting on sex and gender	The gender ratio was 28/48.
Population characteristics	Age of participants varied between 20 and 40 y.o.
Recruitment	Participants were recruited online using the IRSC platform (https://expesciences.risc.cnrs.fr/)
Ethics oversight	All participants gave informed consent. This study complies with all relevant ethical regulations and received formal approval from the INSERM Ethics Committee (CEEI-IRB00003888, decision no 16–333).

Note that full information on the approval of the study protocol must also be provided in the manuscript.

Field-specific reporting

Please select the one below that is the best fit for your research. If you are not sure, read the appropriate sections before making your selection.

☐ Life sciences ☒ Behavioural & social sciences ☐ Ecological, evolutionary & environmental sciences

For a reference copy of the document with all sections, see nature.com/documents/nr-reporting-summary-flat.pdf

Behavioural & social sciences study design

All studies must disclose on these points even when the disclosure is negative.

Study description	This is a quantitative experimental study.
Research sample	All participants were native French speakers, with no reported history of psychiatric or neurological illness. A total of 41 people (28 women; age: mean = 28, SD = 5, min = 20, max = 40) participated in this study.
Sampling strategy	Sample size was chosen according to standard guidelines in the field (with batches of 12 people tested simultaneously).
Data collection	The experiment was developed using Matlab and PsychToolbox, and was conducted entirely in French. Participants were tested at the PRISME platform of the Paris brain Institute, by batches of 12 people (simultaneously), without the presence of the investigator.
Timing	Experiment started on the 20th of January 2018 and ended on the 12th of February 2018.
Data exclusions	No participant was excluded from the study.
Non-participation	No participant dropped.
Randomization	N/A.

Reporting for specific materials, systems and methods

We require information from authors about some types of materials, experimental systems and methods used in many studies. Here, indicate whether each material, system or method listed is relevant to your study. If you are not sure if a list item applies to your research, read the appropriate section before selecting a response.

Materials & experimental systems

n/a	Involved in the study	
☒	☐	Antibodies
☒	☐	Eukaryotic cell lines
☒	☐	Palaeontology and archaeology
☒	☐	Animals and other organisms
☒	☐	Clinical data
☒	☐	Dual use research of concern

Methods

n/a	Involved in the study	
☒	☐	ChIP-seq
☒	☐	Flow cytometry
☒	☐	MRI-based neuroimaging
